# Hypertriglyceridemic waist-to-height ratio and the triple burden of hypertension, diabetes, and dyslipidemia in adults: association and network analysis

**DOI:** 10.3389/fendo.2026.1932657

**Published:** 2026-10-07

**Authors:** Lanqiqi Wu, Jiaoyang Chen, Ting He, Ling Ma, Yin Cheng, Yining Liu, Jinyu Ma, Huitian Tong, Chao Shi

**Affiliations:** 1 School of Public Health, Ningxia Medical University, Yinchuan, China; 2 People’s Hospital of Ningxia Hui Autonomous Region, Ningxia Medical University, Yinchuan, China; 3 Ningxia Institute of Clinical Medicine, People’s Hospital of Ningxia Hui Autonomous Region, Yinchuan, China

**Keywords:** adults, China, diabetes, dyslipidemia, hypertension, hypertriglyceridemic waist-to-height ratio

## Abstract

**Background:**

Hypertension, diabetes, and dyslipidemia frequently co-occur, yet simple screening tools are lacking in resource-limited settings. The hypertriglyceridemic waist-to-height ratio (HTH/WH) phenotype has emerged as a potential indicator, but its utility for identifying the triple burden of hypertension, diabetes, and dyslipidemia remains underexplored, particularly in underdeveloped regions of China.

**Methods:**

This cross-sectional study included 10,796 adults (≥18 years) from the Ningxia Hui Autonomous Region, Northwest China. The HTH/WH phenotype was defined as triglycerides ≥1.7 mmol/L combined with waist-to-height ratio ≥0.5. Multivariable logistic regression was used to estimate adjusted odds ratios (ORs) for the association between the HTH/WH phenotype and coexisting hypertension, diabetes, and dyslipidemia. Mixed graphical models were employed to characterize the network associations among the HTH/WH phenotype, the three cardiometabolic disorders, and modifiable lifestyle factors.

**Results:**

The prevalence of concurrent hypertension, diabetes, and dyslipidemia was 3.99% in Ningxia, exceeding the Northwest China regional average of 2.33%. The HTH/WH phenotype was present in 21.30% of the population and demonstrated a significant, dose-response association with the triple burden (adjusted OR = 5.29, 95% CI: 4.14–6.74), as well as with two diseases (adjusted OR = 5.12, 95% CI: 4.22–6.21) and one disease (adjusted OR = 1.97, 95% CI: 1.77–2.20). Factors independently associated with the triple burden included age ≥40 years, smoking, alcohol consumption, and elevated BMI (≥24 kg/m^2^), while higher education and married status were inversely protective. Mixed graphical modeling revealed the HTH/WH phenotype as a central bridge node (bridge strength = 0.67) within the metabolic disorder network, with strong direct connections to hypertension, diabetes, and dyslipidemia. BMI exhibited the highest bridge centrality (bridge strength = 1.72), followed by smoking (bridge strength = 0.32).

**Conclusions:**

In Ningxia Hui Autonomous Region, concurrent hypertension, diabetes, and dyslipidemia pose a substantial health burden among adults. The HTH/WH phenotype may serve as a practical risk assessment indicator for identifying high-risk individuals with this metabolic multimorbidity.

## Introduction

Cardiovascular diseases (CVD) are the leading cause of death worldwide, accounting for approximately 31% of total mortality (1). Once considered a public health challenge primarily in developed regions, CVD have increasingly become prevalent in low- and middle-income countries due to economic transitions (2). Among CVD, hypertension, diabetes, and dyslipidemia known as the “Three highs”, frequently co-occur as comorbidity (3). This multimorbidity has been described as “the norm rather than the exception,” emphasizing that managing these patients represents the single most significant challenge facing health services in developing regions (4).

In China, the prevalence of hypertension, diabetes, and dyslipidemia has steadily increased over the past decade, exacerbating the national disease burden (5–7). The Chinese Expert Consensus on Standardized Management of the “Three Highs” (2023 Edition) explicitly states that these conditions often cluster as dyads or a triad, with the latter representing the most severe form of metabolic multimorbidity and a major modifiable risk factor for CVD (6, 8). In response, the Healthy China Action Plan (2019–2030) has prioritized integrated management of the “Three highs” to reduce CVD-related illness and death (9).

Identifying high-risk populations for metabolic multimorbidity requires screening tools that are simple, cost-effective, and suitable for resource-limited settings. The hypertriglyceridemic waist phenotype (HTGW), characterized by concurrently elevated waist circumference (WC) and triglyceride (TG), is a practical cardiometabolic risk indicator (10), but its reliance on WC alone is less accurate than waist-to-height ratio (WHtR) in assessing abdominal obesity, especially across diverse age groups and body sizes. The hypertriglyceridemic waist-to-height ratio (HTH/WH) phenotype, combining elevated TG with elevated WHtR, may therefore more useful for identifying people at risk for the triad of hypertension, diabetes, and dyslipidemia (11–13). However, previous studies have largely focused on HTH/WH and single diseases, leaving its association with the simultaneous triple burden unexplored, and the network relationships among HTH/WH, the three disorders, and modifiable factors remain uncharacterized.

The Ningxia Hui Autonomous Region (NHAR), a less developed province in Northwest China, remains understudied regarding metabolic multimorbidity. Its population exhibits a distinctive dietary pattern characterized by high beef and mutton consumption and high saturated fat intake, which differs from the dietary profiles of Eastern and Southern China where seafood, poultry, and vegetables play a more prominent role (14, 15). This dietary structure, combined with limited capacity for comprehensive biochemical testing at primary care facilities (16), creates a context in which the triple-burden prevalence (3.99%) already exceeds the Northwest regional average (2.33%) (17–19). Yet no study has investigated the HTH/WH phenotype as a screening tool for this triple burden in NHAR, nor characterized its network associations with the three diseases and modifiable factors (13, 20).

To address these gaps, this study conducted a cross-sectional study involving adults aged 18 years and older in NHAR. The study aims to: (1) estimate the prevalence of the triple burden of hypertension, diabetes, and dyslipidemia; (2) evaluate the association between HTH/WH phenotype and this triple burden; and (3) explore the network associations among HTH/WH, the three diseases, and key factors through mixed graphical models. The findings from this study will provide evidence for developing targeted and feasible screening and management strategies to address the triple burden in underdeveloped regions such as NHAR.

## Methods

### Study population

This study utilized data from the Ningxia Cardiovascular Disease Survey (NCDS) conducted from January 2020 to December 2021. The detailed design of the NCDS has been described elsewhere (21). Briefly, a 4-stage, stratified cluster sampling method was used to select a regionally representative sample of the general population aged 18 years and older. In the first stage, 9 counties with varied socioeconomic statuses that represent both urban and rural areas were chosen as survey sites (**Supplementary Figure 1**). In the second stage, two towns were selected from each county/district using a simple random sampling method based on the list of towns provided by the local Centers for Disease Control and Prevention. In the third stage, three villages or urban residential communities were selected from each county/district as the study sites using a simple random sampling method. The final stage of sampling was stratified by sex and age distribution according to the China census data from 2010.

A total of 10,803 permanent residents were recruited. Seven were excluded because of incomplete clinical data (missing blood pressure, lipid profile, or blood glucose measurements), leaving 10,796 eligible participants for the final analysis.

### Physical examination data

All study participants were required to undergo physical examinations after fasting for at least 8 hours. Body measurements including height, weight, and WC were collected following standardized protocols, with body mass index (BMI) and WHtR subsequently calculated. After resting for 5 minutes, seated blood pressure in the right arm was measured three times consecutively using a sphygmomanometer (OMRON, HPP-1120U), with the average value recorded. Subsequently, trained investigators collected additional information through face-to-face, one-on-one interviews, including sex, age, educational level, marital status, smoking status, alcohol consumption, and dietary intake frequency. Based on criteria commonly used in previous large-scale cohort studies (22, 23), intake frequency was categorized as “regular” (consumption ≥4 days per week).

### Laboratory test data

Participants were required to conduct an overnight fast before the blood test (at least eight hours but not more than 16 hours). Approximately 5 mL of venous blood samples from participants were collected and processed at the People’s Hospital of Ningxia Hui Autonomous Region. These samples were stored at -80 °C, packaged in dry ice, and transported altogether to the Beijing CIC Medical Laboratory for analysis. An AU5800 Chemistry Analyzer (Beckman Coulter, California, USA) was used to measure the level of total cholesterol (TC), serum low-density lipoprotein cholesterol (LDL-C), serum high-density lipoprotein cholesterol (HDL-C), serum triglycerides (TG), and fasting plasma glucose (FPG). Commercial reagents (Biosino, Beijing, China) were used in this measurement process. The Tosoh Automated Glycohemoglobin Analyzer HLC-723GX (Tosoh Corporation, Tokyo, Japan) was used to measure the level of glycated hemoglobin (HbA1c). All laboratory practices adhered to standardized procedures.

### Definition of key indicators

WHtR was calculated using the formula: WHtR = WC (cm)/height (cm). Based on TG ≥1.7 mmol/L and WHtR ≥ 0.5, participants were divided into two groups: the HTH/WH group and the non-HTH/WH group. Unlike HTGW, which relies on waist circumference alone, HTH/WH uses the waist-to-height ratio, which accounts for height and is more accurate across different body sizes. Hypertension was defined as systolic blood pressure ≥140 mmHg and/or diastolic blood pressure ≥ 90 mmHg and/or current use of antihypertensive medication (24). Diabetes mellitus was defined as fasting plasma glucose ≥ 7.0 mmol/L and/or current use of glucose-lowering drugs (25). Dyslipidemia was defined as meeting any one of the following criteria: total cholesterol ≥ 6.22 mmol/L, triglycerides ≥ 2.26 mmol/L, low-density lipoprotein cholesterol ≥ 4.14 mmol/L, high-density lipoprotein cholesterol < 1.04 mmol/L, and/or current use of lipid-lowering medication, based on the Chinese Guidelines for Lipid Management (25).

### Ethical statement

All participants in this study provided written informed consent prior to enrollment. The study protocol was reviewed and approved by the Ethics Review Committee of the People’s Hospital of Ningxia Hui Autonomous Region (Approval No. 2020-YC-002).

### Statistical analysis

All statistical analyses were performed using R software (version 4.4.3). Descriptive statistics were summarized as mean ± SD or median (IQR) as appropriate, with group comparisons across comorbidity categories using chi-square, Kruskal–Wallis, or Z tests as appropriate.

Multivariable logistic regression models were fitted to estimate associations between the HTH/WH phenotype and multimorbidity outcomes (one, two, or three diseases), with the no-disease group as the reference. The fully adjusted model included age, sex, education, marital status, residence, smoking, alcohol, dietary intakes, and BMI.

Mixed graphical models were estimated using the mgm package (version 1.2.15), with network visualization using qgraph (version 1.9.8), bridge centrality using networktools (version 1.6.0), and regularization via the EBICglasso algorithm (γ = 0.25) (26). In the constructed network, edge weights represent partial correlations between variables after controlling for all other nodes (26, 27); the top 20% of nodes by bridge strength were identified as bridge nodes (26, 28). All tests were two-tailed, and *P*-values less than 0.05 were considered to indicate statistical significance.

## Results

### Baseline characteristics of the participants

The baseline characteristics of the 10,796 participants are summarized in **Table 1**. The cohort included 4,897 males (45.36%) and 5,899 females (54.64%), with a median age of 46 years. Among the participants, 5,973 (55.30%) resided in rural areas, 8,430 (78.08%) were married, and only 4,093 (37.91%) had a normal body mass index (BMI). Significant differences were observed across comorbidity groups in terms of age, gender, residence, educational level, marital status, smoking and drinking habits, BMI categories, and dietary factors (including regular intake of fresh fruits and fried foods) (all *P* < 0.001), with the exception of regular fresh vegetable intake (*P* = 0.143).

**Table 1:** The comorbidity of the three diseases of participants aged 18 years and above in Ningxia Hui Autonomous Region, China.

Variables	Overall (N = 10796)	None (N = 4851)	Just one disease (N = 3715)	Both diseases co-exist (N = 1799)	All three diseases (N = 431)	*P* value
Age, Median (IQR)	46 (33;59)	38 (26;47)	50 (38;51)	57 (48;58)	61 (53;63)	<0.001^b^
Gender, n (%)						<0.001^a^
Male	4897 (45.36)	1799 (37.09)	1882 (50.66)	1004 (55.81)	212 (49.19)	
Female	5899 (54.64)	3052 (62.91)	1833 (49.34)	795 (44.19)	219 (50.81)	
Residence, n (%)						<0.001^a^
Urban	4823 (44.67)	2034 (41.93)	1687 (45.41)	888 (49.36)	214 (49.65)	
Rural	5973 (55.33)	2817 (58.07)	2028 (54.59)	911 (50.64)	217 (50.35)	
Education, n (%)						<0.001^a^
Primary schools and below	3801 (35.21)	1178 (24.28)	1506 (40.54)	882 (49.03)	235 (54.52)	
Middle school	3071 (28.45)	1289 (26.57)	1135 (30.55)	508 (28.24)	139 (32.25)	
High school or above	3924 (36.65)	2384 (49.14)	1074 (28.91)	409 (22.73)	57 (13.23)	
Marital status, n (%)						<0.001^a^
Married	8430 (78.08)	3452 (71.16)	3073 (82.72)	1547 (85.99)	358 (83.06)	
Unmarried	2366 (21.92)	1399 (28.84)	642 (17.28)	252 (14.01)	73 (16.94)	
Smoking, n (%)						<0.001^a^
No	8262 (76.53)	3919 (80.79)	2742 (73.81)	1271 (70.65)	330 (76.57)	
Yes	2534 (23.47)	932 (19.21)	973 (26.19)	528 (29.35)	101 (23.43)	
Drinking, n (%)						<0.001^a^
No	8539 (79.09)	3916 (80.73)	2867 (77.17)	1394 (77.49)	362 (83.99)	
Yes	2257 (20.91)	935 (19.27)	848 (22.83)	405 (22.51)	69 (16.01)	
BMI grouping, n (%)						<0.001^a^
< 24	4453 (41.25)	2853 (58.81)	1196 (32.19)	347 (19.29)	57 (13.23)	
≥ 24	6343 (58.75)	1988 (41.19)	2519 (67.81)	1452 (80.71)	374 (86.77)	
HTH/WH phenotypes, n (%)						<0.001^a^
No	8494 (78.68)	4583 (94.48)	2827 (76.10)	939 (52.20)	145 (33.64)	
Yes	2302 (21.32)	268 (5.52)	888 (23.90)	860 (47.80)	286 (66.36)	
Regular fresh vegetable intake						0.143^a^
No	654 (6.06)	335 (6.91)	206 (5.55)	94 (5.23)	19(4.41)	
Yes	10142 (93.94)	4516 (93.09)	3509 (94.45)	1705 (94.77)	412 (95.59)	
Regular fresh fruits intake						<0.001^a^
No	1150 (10.65)	374 (7.71)	441 (11.87)	258 (14.34)	77(17.87)	
Yes	9694 (89.35)	4477 (92.29)	3274 (88.13)	1541 (85.66)	354 (82.13)	
Regular fried foods intake						<0.001^a^
No	8007 (74.17)	3505 (72.25)	2776 (74.72)	1390 (77.27)	336(77.96)	
Yes	2789 (25.83)	1346 (27.75)	939 (25.28)	409 (22.73)	95 (22.04)	
Laboratory data, Mean ± SD						
HbA1c (%)	5.64 ± 0.79	5.38 ± 0.38	5.67 ± 0.76	5.94 ± 0.94	7.11 ± 1.43	<0.001^c^
FPG (mmol/L)	5.71 ± 1.47	5.24 ± 0.56	5.76 ± 1.47	6.18 ± 1.58	8.67 ± 3.10	<0.001^c^
TG (mmol/L)	1.48 ± 1.35	1.00 ± 0.46	1.65 ± 1.33	2.12 ± 1.38	3.46 ± 3.42	<0.001^c^
TC (mmol/L)	4.27 ± 0.97	4.05 ± 0.76	4.37 ± 1.01	4.53 ± 1.15	4.82 ± 1.38	<0.001^c^
HDL-C (mmol/L)	1.26 ± 0.28	1.35 ± 0.24	1.21 ± 0.29	1.15 ± 0.26	1.09 ± 0.26	<0.001^c^
LDL-C (mmol/L)	2.59 ± 0.78	2.41 ± 0.64	2.69 ± 0.80	2.79 ± 0.90	2.88 ± 1.03	<0.001^c^
Mean SBP (mmHg)	130.24 ± 20.05	117.79 ± 10.79	135.39 ± 19.93	149.63 ± 16.61	151.23 ± 17.36	<0.001^c^
Mean DBP (mmHg)	80.94 ± 11.17	75.10 ± 7.58	83.64 ± 11.09	89.86 ± 10.63	88.91 ± 11.37	<0.001^c^

BMI, body mass index; HbA1c, glycated hemoglobin; FPG, fasting plasma glucose; TG, triglycerides; TC, total cholesterol; HDL-C, high-density lipoprotein cholesterol; LDL-C, low-density lipoprotein cholesterol; SBP, systolic blood pressure; DBP, diastolic blood pressure.

a *P* values were obtained from chi-square test.

b *P* value was obtained from Kruskal–Wallis rank-sum test.

c *P* values for laboratory measurements were obtained using the Z test (a normal-approximation test for large-sample comparisons).

Notably, the prevalence of the HTH/WH phenotype markedly increased with the number of comorbidities: it was present in 5.52% of individuals without comorbidities, 23.90% of those with one comorbidity, 47.80% of those with two co-existing diseases, and 66.36% among participants with all three diseases (*P* < 0.001). Laboratory measures also displayed significant trends across the comorbidity groups. Levels of HbA1c, fasting plasma glucose (FPG), triglycerides (TG), total cholesterol (TC), and low-density lipoprotein cholesterol (LDL-C) progressively increased with the accumulation of comorbidities, while high-density lipoprotein cholesterol (HDL-C) levels decreased (all *P* < 0.001). Similarly, both systolic and diastolic blood pressure values were significantly higher in groups with a greater number of comorbidities (*P* < 0.001).

### Age and gender distribution of the prevalence of three diseases

In the single comorbidity pattern, the prevalence rates exhibited distinct age-dependent distributions between genders. Among males, the prevalence increased rapidly in the 18–30 age group, followed by a slight decline in the 30–40 age group, and subsequently rose again in the 40–50 age group, peaking around age 50. In contrast, the prevalence among females remained relatively stable until age 50; after this age, it increased markedly and subsequently surpassed that of males. For both dual and triple comorbidity patterns, the prevalence in both males and females exhibited a consistent upward trend with advancing age. Within the 18–50 age range, females consistently demonstrated lower comorbidity prevalence compared to males. However, this gender disparity began to diminish after age 40. Notably, by age 50, the prevalence among females exceeded that of males, indicating a significant reversal (**Figure 1**).

**Figure 1:**
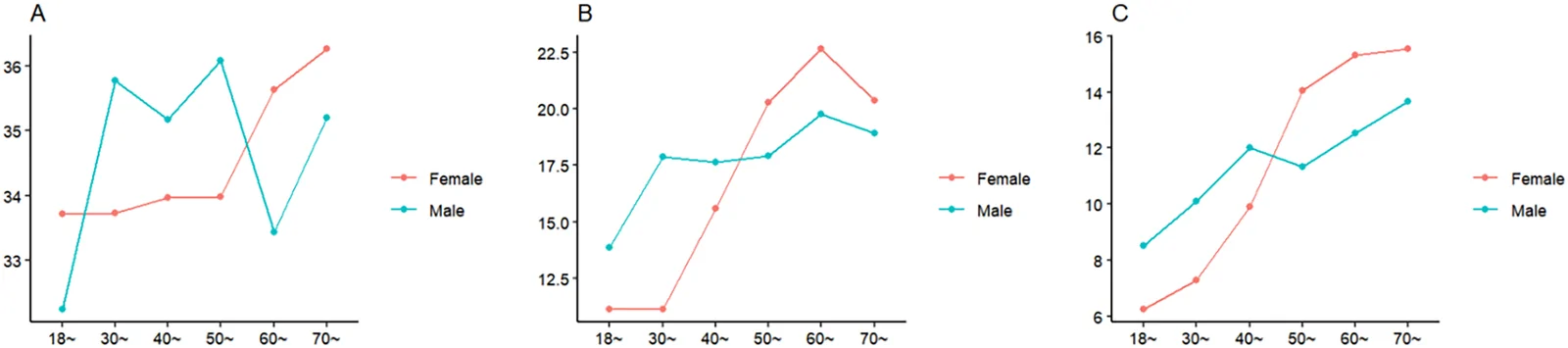
Age and gender distribution of the prevalence of individuals with three coexisting diseases in 10,796 individuals from Ningxia Hui Autonomous Region, China. **(A)** Just one disease, **(B)** Both diseases co-exist, **(C)** All three disease.

### Factors associated with the prevalence of three diseases

Multivariable logistic regression was employed to assess factors associated with multimorbidity, and the results are detailed in **Table 2**. For the triple-comorbidity outcome (all three diseases), the fully adjusted model identified age ≥40 years (adjusted OR range: 1.49-1.95), smoking (adjusted OR = 1.53, 95% CI: 1.32-1.78), alcohol consumption (adjusted OR = 1.19, 95% CI: 1.04-1.39), and BMI ≥24 kg/m^2^ (adjusted OR = 2.97, 95% CI: 2.60-3.40) as factors significantly associated with increased risk. Conversely, higher education level (adjusted OR range: 0.48-0.71) and married status (adjusted OR = 0.80, 95% CI: 0.66-0.96) were associated with lower odds. Notably, urban residence (adjusted OR = 1.04, 95% CI: 0.91-1.18) and inadequate fresh fruit intake (adjusted OR = 0.97, 95% CI: 0.80-1.18) were not significantly associated with triple comorbidity in the fully adjusted model. The HTH/WH phenotype was significantly associated with the coexistence of all three diseases (adjusted OR = 5.29, 95% CI: 4.14-6.74), with two diseases (adjusted OR = 5.12, 95% CI: 4.22-6.21), and with one disease (adjusted OR = 1.97, 95% CI: 1.77-2.20) (all *P* < 0.001).

**Table 2:** Risk factors for co-occurrence of three diseases.

Variables	Just one disease		Both diseases co-exist		All three diseases	
	Crude OR (95% CI)	Adjusted OR (95% CI)	Crude OR (95% CI)	Adjusted OR (95% CI)	Crude OR (95% CI)	Adjusted OR (95% CI)
Age						
18~	Reference	Reference	Reference	Reference	Reference	Reference
30~	1.09 (0.96 to 1.25)	1.08 (0.94 to 1.23)	1.13 (0.94 to 1.35)	1.09 (0.91 to 1.32)	1.12 (0.89 to 1.41)	1.08 (0.86 to 1.37)
40~	1.08 (0.95 to 1.23)	1.04 (0.91 to 1.19)	1.41 (1.18 to 1.68)	1.33 (1.12 to 1.60)	1.57 (1.26 to 1.94)	1.49 (1.19 to 1.85)
50~	1.13 (0.99 to 1.28)	1.05 (0.93 to 1.20)	1.58 (1.33 to 1.88)	1.40 (1.17 to 1.67)	1.72 (1.39 to 2.13)	1.52 (1.23 to 1.89)
60~	1.06 (0.92 to 1.23)	0.99 (0.86 to 1.16)	2.02 (1.67 to 2.43)	1.81 (1.49 to 2.18)	2.18 (1.74 to 2.73)	1.95 (1.55 to 2.45)
70~	1.14 (0.98 to 1.32)	1.07 (0.92 to 1.23)	1.72 (1.42 to 2.09)	1.52 (1.25 to 1.85)	2.13 (1.70 to 2.68)	1.89 (1.50 to 2.38)
Gender, n (%)						
Female	Reference	Reference	Reference	Reference	Reference	Reference
Male	1.01 (0.94 to 1.10)	1.01 (0.93 to 1.09)	1.10 (0.99 to 1.22)	1.08 (0.98 to 1.21)	1.04 (0.92 to 1.18)	1.02 (0.89 to 1.15)
Residence, n (%)						
Rural	Reference	Reference	Reference	Reference	Reference	Reference
Urban	1.05 (0.97 to 1.13)	1.06 (0.97 to 1.15)	1.26 (1.13 to 1.39)	1.29 (1.16 to 1.44)	1.05 (0.93 to 1.19)	1.04 (0.91 to 1.18)
Education, n (%)						
Primary schools and below	Reference	Reference	Reference	Reference	Reference	Reference
Middle school	0.89 (0.81 to 0.99)	0.84 (0.76 to 0.93)	0.65 (0.58 to 0.73)	0.58 (0.51 to 0.66)	0.80 (0.69 to 0.92)	0.71 (0.61 to 0.82)
High school or above	0.57 (0.52 to 0.63)	0.62 (0.56 to 0.69)	0.38 (0.34 to 0.44)	0.44 (0.39 to 0.51)	0.42 (0.36 to 0.49)	0.48 (0.41 to 0.57)
Marital status, n (%)						
Unmarried	Reference	Reference	Reference	Reference	Reference	Reference
Married	0.65 (0.58 to 0.71)	0.84 (0.75 to 0.93)	0.53 (0.46 to 0.61)	0.79 (0.67 to 0.92)	0.80 (0.66 to 0.96)	0.80 (0.66 to 0.96)
Smoking, n (%)						
No	Reference	Reference	Reference	Reference	Reference	Reference
Yes	1.25 (1.14 to 1.38)	1.19 (1.07 to 1.32)	1.45 (1.29 to 1.62)	1.49 (1.30 to 1.70)	1.58 (1.39 to 1.81)	1.53 (1.32 to 1.78)
Drinking, n (%)						
No	Reference	Reference	Reference	Reference	Reference	Reference
Yes	1.19 (1.08 to 1.31)	1.17 (1.05 to 1.30)	1.12 (0.99 to 1.27)	1.03 (0.89 to 1.19)	1.33 (1.15 to 1.53)	1.19 (1.04 to 1.39)
Fresh vegetables						
Yes	Reference	Reference	Reference	Reference	Reference	Reference
No	1.15 (0.97 to 1.37)	0.93 (0.77 to 1.10)	1.21 (0.97 to 1.52)	0.91 (0.71 to 1.14)	1.19 (0.91 to 1.56)	0.93 (0.70 to 1.22)
Fresh fruit						
Yes	Reference	Reference	Reference	Reference	Reference	Reference
No	0.83 (0.73 to 0.94)	1.05 (0.93 to 1.21)	0.65 (0.56 to 1.01)	1.26 (1.07 to 1.47)	0.84 (0.70 to 1.02)	0.97 (0.80 to 1.18)
Fried foods						
No	Reference	Reference	Reference	Reference	Reference	Reference
Yes	0.96 (0.87 to 1.05)	0.96 (0.87 to 1.05)	0.81 (0.72 to 0.92)	0.85 (0.75 to 0.97)	0.85 (0.74 to 0.98)	0.86 (0.74 to 0.99)
BMI grouping, n (%)						
< 24	Reference	Reference	Reference	Reference	Reference	Reference
≥ 24	1.64 (1.51 to 1.79)	1.66 (1.53 to 1.80)	3.21 (2.83 to 3.64)	3.02 (2.70 to 3.37)	3.34 (2.85 to 3.90)	2.97 (2.60 to 3.40)
HTH/WH phenotypes, n (%)						
No	Reference	Reference	Reference	Reference	Reference	Reference
Yes	2.34 (2.14 to 2.56)	1.97 (1.77 to 2.20)	8.11 (6.81 to 9.67)	5.12 (4.22 to 6.21)	8.19 (6.55 to 10.24)	5.29 (4.14 to 6.74)

BMI, body mass index.

### Network associations among modifiable factors, HTH/WH phenotype, and cardiometabolic disorders

In **Figure 2**, there are strong positive correlations among the three diseases. Additionally, the HTH/WH phenotype exhibits strong positive connections with hypertension, diabetes, and dyslipidemia, with edge weights of 0.56, 0.46, and 0.50, respectively. Among modifiable factors, BMI has strong positive correlations with hypertension, diabetes, dyslipidemia, and the HTH/WH phenotype, with edge weights of 0.41, 0.28, 0.32, and 0.71, respectively. Moreover, the intake of fresh fruits has relatively strong negative connections with the HTH/WH phenotype and the three diseases. The edge weights are presented in **Supplementary Figure 2**. From the bridge strength plot, among the influencing factors, BMI has the highest bridge centrality (bridge strength = 1.72), followed by smoking (bridge strength = 0.32). The bridge strength of the HTH/WH phenotype is 0.67. For the three diseases, the bridge centrality values, in descending order, are dyslipidemia (bridge strength = 0.57), hypertension (bridge strength = 0.27), and diabetes (bridge strength = 0.01) (all *P* < 0.001).

**Figure 2:**
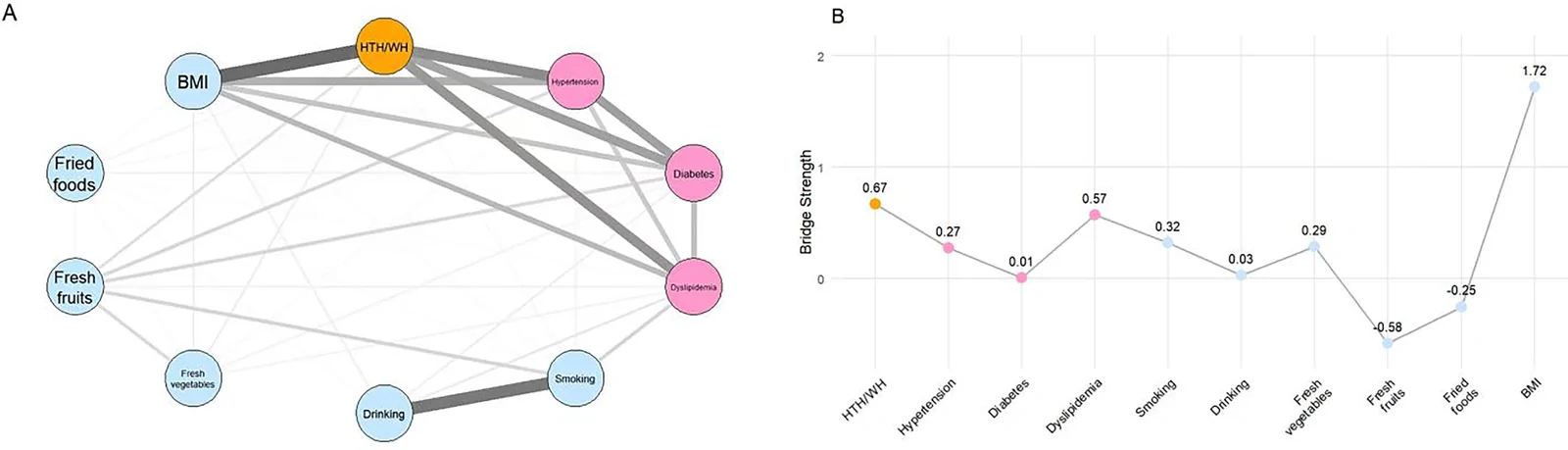
Network analysis and bridge strength of hypertension, diabetes, dyslipidemia, HTH/WH, and associated influencing factors in Ningxia Hui Autonomous Region, China. (n=10,796). **(A)** Network analysis with Mixed-Effects Model Figure. The width of the gray edges represents the strength of associations; all three diseases, HTH/WH, and influencing factors were included as categorical variables, **(b)** Bridge strength.

## Discussion

Our study yielded several significant findings regarding the cardiometabolic health profile of adults in the NHAR. First, this study observed a substantial burden of concurrent hypertension, diabetes, and dyslipidemia, with a prevalence of 3.99%. Second, the HTH/WH phenotype was highly prevalent (21.30%) and demonstrated a robust, dose-response association with the co-occurrence of these metabolic disorders. Third, and most notably, mixed graphical modeling identified the HTH/WH phenotype as a central bridge node within the metabolic disorder network, underscoring its pivotal interconnecting role between hypertension, diabetes, and dyslipidemia.

The co-prevalence of any two cardiometabolic disorders in our population reached 16.66%, a rate considerably higher than those previously reported in Southern China (12.79%) (29)and Xinjiang (11.60%) (30). Notably, the prevalence of full triple comorbidity (3.99%) not only surpassed the average reported in Northwest China (2.33%) for the period between 2013 and 2018 (17, 18), but also approached that observed among Indian adults (6.36%) (31). Although the absolute prevalence of 3.99% is less than 5%, it exceeds the Northwest China regional average by more than 70%, translating to nearly 290,000 adults among Ningxia’s population of approximately 7.2 million who are simultaneously bearing the triple metabolic burden. This represents a considerable strain on local primary healthcare resources and chronic disease management capacity, and thus carries important early-warning value for regional public health prevention and control efforts. This elevated burden positions the NHAR, an underdeveloped area of China, as a region of particular concern. It was postulated postulated that characteristic local dietary patterns, marked by a high intake of red meat (especially beef and mutton), rich in saturated fats, may be associated with this adverse metabolic profile (15, 32). Such a diet is often imbalanced and is a well-established risk factor for cardiometabolic diseases.

Gender-stratified analyses revealed distinct patterns. For single-disease prevalence, males exhibited an increase in the 18–30 age group, potentially attributable to the early adoption of unhealthy lifestyle habits prevalent in this demographic, such as diets high in salt and fat, smoking, and excessive alcohol consumption (33, 34). Furthermore, professional pressures, psychological stress, and inadequate sleep among young males may exacerbate metabolic dysregulation (35, 36). In contrast, the prevalence of multiple disease patterns (both dual and triple comorbidities) increased progressively with age in all genders, consistent with the natural decline in physical activity, immune function (37), and potentially lower health literacy regarding chronic disease management among older adults with limited education (38). The gender gap observed in this study, where women aged 18–50 had lower comorbidity rates than men but the gap narrowed after 40 and reversed by 50, likely reflects the loss of cardioprotective effects after menopause (39). Beyond age and sex, our findings align with existing literature, identifying smoking, alcohol consumption, and elevated BMI (≥24 kg/m^2^) as factors consistently associated with increased multimorbidity risk across outcome levels, while higher education level and married status were associated with reduced risk (40–42). Notably, urban residence and inadequate fresh fruit intake were significantly associated with two-disease comorbidity but not with triple comorbidity in the fully adjusted model.

The HTH/WH phenotype serves as a simple yet effective risk indicator for assessing abdominal obesity and metabolic disorders (43). Its prevalence of 21.30% in NHAR adults significantly exceeds figures from the China Health and Retirement Longitudinal Study (15.93%) (44) and the global adult population estimate (18.00%) (45). The strong, graded association between the HTH/WH phenotype and the number of co-occurring metabolic disorders, even after extensive multivariable adjustment, underscores its utility as a composite risk indicator. This association is biologically plausible, as the HTH/WH phenotype is a well-established surrogate for insulin resistance and visceral adiposity, which are factors closely associated with hypertension, dysglycemia, and atherogenic dyslipidemia.

A key innovation of our study was the application of mixed graphical models to extend beyond traditional pairwise associations. The network analysis revealed strong interconnections among hypertension, diabetes, and dyslipidemia, visually depicting the association patterns that underlie their frequent co-occurrence (26, 46, 47). Within this network, the HTH/WH phenotype emerged as a critical bridge node (bridge strength = 0.67), with strong edges directly connecting it to all three disorders. This positions the HTH/WH phenotype not merely as a correlate, but as a potential central hub in the shared metabolic network. It should be emphasized that network analysis reveals partial correlations among variables rather than causal relationships or biological mechanisms; the identified associations may reflect common underlying pathways such as systemic inflammation and insulin resistance, and should be further validated through future longitudinal studies or basic experimental research. As anticipated, BMI was the node with the highest bridge strength, reaffirming its paramount role in cardiometabolic health across diverse populations (48–50). Conversely, daily fresh fruit intake exhibited an inverse association within the network, highlighting its potential role in mitigating metabolic risk (51).

### Strengths and limitations

The major strengths of our study include its large, population-based sample, the use of standardized measurements, and the innovative application of network analysis to elucidate the complex relationships between a simple phenotypic marker and multiple metabolic diseases. However, several limitations warrant consideration. First, the cross-sectional design precludes any causal inferences regarding the relationship between the HTH/WH phenotype and disease onset; nor can the study truly evaluate the long-term predictive value of HTH/WH. Future prospective cohort studies employing methods such as ROC curve analysis are warranted to validate the actual predictive performance of the HTH/WH phenotype. Second, assessing HTH/WH still requires a fasting triglyceride test, so it is not entirely non-invasive or independent of laboratory work. Its advantage in resource-limited settings is that it streamlines screening and reduces the number of tests needed, requiring only one biochemical marker plus WC and height, rather than replacing biochemical testing altogether. Third, although we used multistage stratified cluster sampling, we did not incorporate sampling weights or clustering effects in the statistical analyses, which may have underestimated standard errors. Our findings should be interpreted with this in mind, and future studies using weighted approaches would help validate the results. Fourth, dietary intake and other lifestyle factors were self-reported, which can introduce recall bias. Finally, residual confounding from unmeasured variables may remain.

## Conclusion

This study revealed a notably high prevalence of concurrent hypertension, diabetes mellitus, and dyslipidemia among adults in the NHAR. The HTH/WH phenotype was widely distributed in this population and demonstrated a significant association with an elevated odds of these co-occurring metabolic disorders. As a practical risk assessment indicator that requires only triglyceride testing combined with anthropometric measurements, the HTH/WH phenotype showed potential for identifying high-risk individuals with multiple metabolic abnormalities, thereby assisting primary care physicians in prioritizing patients for further comprehensive evaluation and intervention.

## Data Availability

Data are not publicly available due to ethical and privacy restrictions imposed by the Institutional Review Board of the People’s Hospital of Ningxia Hui Autonomous Region (Approval No. 2020-YC-002), which protect participant confidentiality. The dataset contains potentially identifying information (e.g., age, sex, ethnicity, residential region), and participants provided written informed consent only for use of their data in this specific study, not for public deposition.
