# Supplementary material for: Hypertriglyceridemic waist-to-height ratio and the triple burden of hypertension, diabetes, and dyslipidemia in adults: association and network analysis
Source: Front Endocrinol (Lausanne). 2026 Oct 7;17:1932657. doi: 10.3389/fendo.2026.1932657 (PMC13683101; doi:10.3389/fendo.2026.1932657)
Supplement: Supplementary file 1 [file Supplementaryfile1.docx]

**Supplementary Figure 1.** Distribution of study area for cardiovascular disorders and the related risk factors survey in Ningxia Hui Autonomous Region, 2020-2021.

**Supplementary Figure 2.** Polychoric Correlation Heatmap of HTH/WH, Cardiometabolic Diseases, and Modifiable Lifestyle Factors.
